# Machine learning based suicide prediction and development of suicide vulnerability index for US counties

**DOI:** 10.1038/s44184-022-00002-x

**Published:** 2022-06-01

**Authors:** Vishnu Kumar, Kristin K. Sznajder, Soundar Kumara

**Affiliations:** 1 Department of Industrial and Manufacturing Engineering, The Pennsylvania State University, University Park, PA USA; 2 Department of Public Health Sciences, The Pennsylvania State University College of Medicine, Hershey, PA USA

**Keywords:** Public health, Preventive medicine

## Abstract

Suicide is a growing public health concern in the United States. A detailed understanding and prediction of suicide patterns can significantly boost targeted suicide control and prevention efforts. In this article we look at the suicide trends and geographical distribution of suicides and then develop a machine learning based US county-level suicide prediction model, using publicly available data for the 10-year period from 2010–2019. Analysis of the trends and geographical distribution of suicides revealed that nearly 25% of the total counties experienced at least a 10% increase in suicides from 2010 to 2019, with about 12% of total counties exhibiting an increase of at least 50%. An eXtreme Gradient Boosting (XGBoost) based machine learning model was used with 17 unique features for each of the 3140 counties in the US to predict suicides with an *R*^2^ value of 0.98. Using the SHapley Additive exPlanations (SHAP) values, the importance of all the 17 features used in the prediction model training set were identified. County level features, namely *Total Population, % African American Population, % White Population, Median Age* and *% Female Population* were found to be the top 5 important features that significantly affected prediction results. The top five important features based on SHAP values were then used to create a Suicide Vulnerability Index (SVI) for US Counties. This newly developed SVI has the potential to detect US counties vulnerable to high suicide rates and can aid targeted suicide control and prevention efforts, thereby making it a valuable tool in an informed decision-making process.

## Introduction

**Fig. 1:**
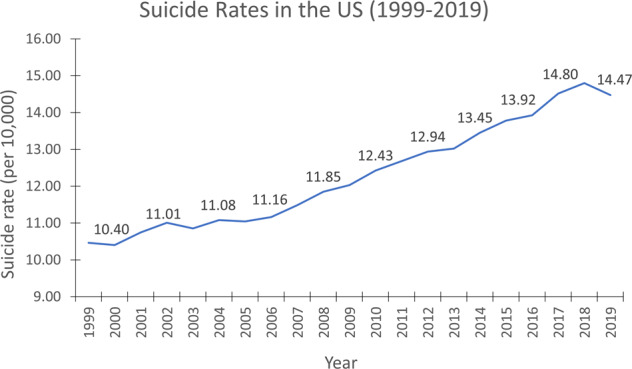
Suicide rates in the US (1999–2019) per 10,000. Data Source: Centers for Disease Control and Prevention (CDC), USA.

Suicide is the act of intentionally causing self-death and is a large and growing public health problem^1^. Suicide has become one among the top ten leading causes of death in the US, with more than 47,500 deaths in the year 2019^2^. World Health Organization (WHO) statistics^3^ reveal that the US ranks in the 24^th^ position among all countries with respect to suicide rates, much higher than countries such as India (38^th^ position), where the total population is at least 4 times that of the US. According to the Centers for Disease Control and Prevention (CDC), suicides rates in the US have increased by 33% between 1999 and 2019, with a small dip during 2019^2,4^. Figure 1 shows the suicide trend in the US between 1999 and 2019.

Numerous strategies and resources^5–10^ have been identified and developed in the past to prevent suicides; however, most of these interventions are for large geographic areas at either the state level or national level. While US state level trends in suicides, contextual factors, and prediction models are well documented^11–15^, these may not necessarily reflect the suicide trends of a particular region or geographic area. Since US counties are the smallest possible geographical classification available in the CDC Wonder Database, county level suicides have been studied in the past^16–20^. However, the previously published papers do not explore the possibility of detailed suicide prediction, the impact of contextual factors on suicide rates, or identify regions prone to increased suicide rates at the county level. Therefore, there exists an urgent need for a detailed analysis in this area. Such a detailed investigation at the smallest possible level can significantly boost targeted suicide control and prevention efforts.

This paper tries to bridge this gap by first analyzing US county level suicides for a 10-year period from 2010–2019 and then developing a suicide prediction model using machine learning techniques. The machine learning model described in this paper used 17 unique county level characteristic data, grouped into three categories: Demographics, Socio-economic Factors, and Health, to predict suicide rates as explained in detail in the Methods section. The importance of these factors on the prediction results are then identified and used to generate a Suicide vulnerability index (SVI) for all 3140 US Counties. The SVI developed in this paper accurately identifies the counties prone to high suicide rates and those counties susceptible to low suicide rates. The performance of the proposed SVI tool was validated using the data reported by publicly available data sources and study reports. It is hoped that the proposed SVI would not only act as a resource to identify counties or regions at the smallest possible level that are prone to high suicide rates, but also prove to be useful in effectively allocating resources and providing the necessary help, and support to boost targeted suicide control and preventing efforts.

## Methods

### Data description

This work used publicly available data to predict county level suicide rates and to develop the SVI. Suicide data and county characteristics data for 3140 counties in the US between 2010 to 2019 were extracted. A detailed description of the data and the sources are described in this section.

The county level suicide data for the period 2010 to 2019 was extracted from the Multiple Cause of Death dataset of the CDC Wide-ranging ONline Data for Epidemiologic Research (WONDER) database^21^. Suicide deaths, from the cause of deaths, were identified from this database using the International Classification of Disease, Tenth Revision (ICD-10) codes X60-64 (intentional self-harm). The ICD-10 was specifically chosen since the CDC Wonder Database classifies causes of death in the US from 1999-present under the 10^th^ revision^22^. Based on a literature search^23–28^ and availability of reliable data, 17 characteristics, that were considered relevant to suicide deaths, were identified. These characteristics were then grouped into three categories namely: Demographics, Socio-economic Factors, and Health.

The “Demographics” group describes county level features such as the total population estimates (Population), population composition including sex distribution (% Female), ethnic distribution (% White, % African American, % Other races) and median age of the population (Median Age). To study the impact of prominent races in the US such as Whites and Black/African Americans on suicide prediction and SHAP summary, they were isolated and listed as independent factors: %White and %African American. Other races such as Asian American, American Indian/Alaska Native, and Native Hawaiian/Pacific Islander were found to be less prominent in many counties and hence were combined into one group: % Other races. The data for the features in this group were extracted from the US Census Bureau: County Population by Characteristics: 2010–2019^29^. The “Socio-economic Factors” groups encompasses the prominent social and economic factors of counties including Median Income, % Poverty, % Population Unemployed, % Some College (Population who attended some college), % Single Parent Household, Social Association Rate (The amount of community involvement measured using the number of membership associations) and Violent Crime Rate. The median household income (Median Income) and percent population in poverty (% Poverty) estimate data were obtained from the US Census Bureau: Small Area Income and Poverty Estimates (SAIPE)^30^. US Bureau of Labor Statistics County Data Tables^31^ provided the percent population unemployed (% Unemployment) for each county. Data regarding the social factors such as the Social Association Rate (per 10,000 persons), Single-parent Households (as a percent of total population), % Some College, Violent Crime Rate (per 100,000 persons) were published publicly by the County Health Rankings & Roadmaps, a program of the University of Wisconsin Population Health Institute^32^. Finally, the “Health” group captures features such as the Opioid Dispensing Rate (per 100 persons) extracted from the CDC US County Opioid Dispensing Rates^33^, Percent population not insured (% Uninsured) obtained from the US Census Bureau: Small Area Health Insurance Estimates (SAHIE)^34^. The County Health Rankings & Roadmaps, a program of the University of Wisconsin Population Health Institute^32^ provided data on the Mentally Unhealthy Days (in number of days) and excessive drinkers expressed as a percentage of total population (% Excessive Drinking). Table 1 lists the different county level factors used under the three categories with their data sources.

**Table 1:** County level factors grouped under the three categories with their data sources, 2010–2019.

County Level Factor	Data Source
Demographics Category	
Population (in thousands)	US Census CPC
% Female	US Census CPC
% White	US Census CPC
% African American	US Census CPC
% Other races	US Census CPC
Median Age (in years)	US Census CPC
Socio-economic Factors Category	
Median Income (in thousand $)	US Census SAIPE
% Poverty	US Census SAIPE
% Unemployed	BLS LAU
% Some College	CHRR
Social Association Rate (per 10,000 persons)	CHRR
% Single-Parent Households	CHRR
Violent Crime Rate (per 100,000 persons)	CHRR
Health Category	
Opioid Dispensing Rate (per 100 persons)	CDC ODR
% Uninsured	US Census SAHIE
Mentally Unhealthy Days (in number of days)	CHRR
% Excessive Drinking	CHRR

*CPC* County Population by Characteristics; *SAIPE* Small Area Income and Poverty Estimates; *BLS LAU* U.S. Bureau of Labor Statistics. Local Area Unemployment Statistics; *CHRR* County Health Rankings & Roadmaps; *CDC ODR* Centers for Disease Control and Prevention US Opioid Dispensing Rate Map; *SAHIE* Small Area Health Insurance Estimates.

### Prediction model description

Details regarding the machine learning based suicide prediction model, its development and features are discussed in this section. This work essentially involved the prediction of suicides by modeling a relationship between suicides (dependent variable) and the 17 county level factors (independent variables). Since the work predicted a numerical value (suicides), it was modeled as a Regression based Predictive Modeling problem and the extreme gradient boosted decision tree (XGBoost) regressor^35^ was used for prediction. XGBoost was specifically selected since Tree SHAP^36^ has been integrated into the XGBoost and hence can be seamlessly used to compute SHAP values^37^. The software used for the model development and analysis was Python, along with its libraries.

The dataset was split into a training dataset and a testing dataset in the ratio 80:20. The model was calibrated using the training dataset and the accuracy was assessed using the testing dataset. The hyperparameters of the model was tuned using a grid search approach along with 10-fold cross validation.

### Explainability using feature importance

One can possibly argue that not all the factors in the prediction model contributed equally to determining the suicide rates of a particular county. Therefore, there is a strong need to investigate and identify which variables affected the prediction results and quantify their contribution^37^. This is often materialized using the importance score. The importance score gives a rough idea about the significance of each feature in the construction of the prediction model. The more a feature is used by the model in its decision making, the higher is its importance score^38^. Since the feature importance values is computed for each attribute explicitly, the attributes can be easily ranked and compared. Even though XGBoost model can provide estimates of the importance scores for the attributes used with the help of its inbuilt function, they are found to contradict with each other when the parameter “importance types” are changed^37^. The XGBoost Documentation^39^ provides a detailed explanation of the parameter “important types”. Hence this work uses SHapley Additive exPlanations (SHAP) values to identify the impact of each feature on the suicide prediction result unambiguously.

SHAP is based on game theory and can be used to explain the output of any machine learning model^40^. Essentially, SHAP values explore the impact of each feature by comparing the prediction results with and without that feature^41^. In this work, the SHAP values are computed using the SHAP package in Python.

### Development of SVI

The feature importance section above described the process to identify the significance of each feature in determining the output. Some features such as the total population, Median Age, % African American population etc. have high influences on the output than the others. Using the top five features that significantly affect the suicide rates, this work proposes the development of a SVI for all the US counties. Similar to the Social Vulnerability Index^42,43^, SVI refers to the socioeconomic, demographic and other factors that have a high impact on the resilience of counties towards suicides. In other words, counties with high SVI are prone to more suicides than the counties with low SVI. Hence SVI can help in targeted suicide control and prevention efforts.

To develop the SVI for US counties, the top 5 features and their impact on prediction results as obtained from Feature Importance are used as inputs. The features which had positive impact on the output (Population, % White Population and Median Age) were ranked from highest to lowest and the rest (% African American Population and % Female Population) which had a negative impact on the output were ranked lowest to highest. A percentile rank^43^ was then calculated for each of these features. Percentile rank is the percentage of scores in a distribution which are below the score under consideration. For example, if a value has a percentile rank = 0.95, it means that 95% of the values in that distribution are below the value under consideration. Once the percentile rank was assigned to all the five features for each county, the SVI of the county was calculated as a weighted function of the variables under consideration.

In other words,

1 $${{{\mathrm{SVI = }}}}\mathop {\sum}\limits_{{{{\mathrm{(i = 1)}}}}}^{{{\mathrm{p}}}} {\mathrm{W}}_{\mathrm{i}}{\mathrm{X}}_{\mathrm{i}}$$

where,

W_i_ = Weighted contribution of each of the 5 features under consideration

X_i_ = Percentile score of each feature of the county under consideration

i = 1, 2, ……. *p* number of features (in this case *p* = 5)

Note that the weighted contribution of each of the five features were interpreted from the average of SHAP value magnitudes^44^ for the corresponding features. The SVI score for each county was then calculated on a scale of 0–1, with 1 indicating high vulnerability and 0 indicating low vulnerability.

## Results

This section is divided into four parts. The first part describes the trends and geographical distribution of county level suicide rates in the US based on the data between 2010–2019. Next, the suicide prediction model results are discussed. The feature importance results obtained from the prediction model are then analyzed and explained in the third part. Finally, the feature importance results are used to develop a county level SVI, and the results are discussed.

### Trends and geographical distribution of county level suicides

**Fig. 2:**
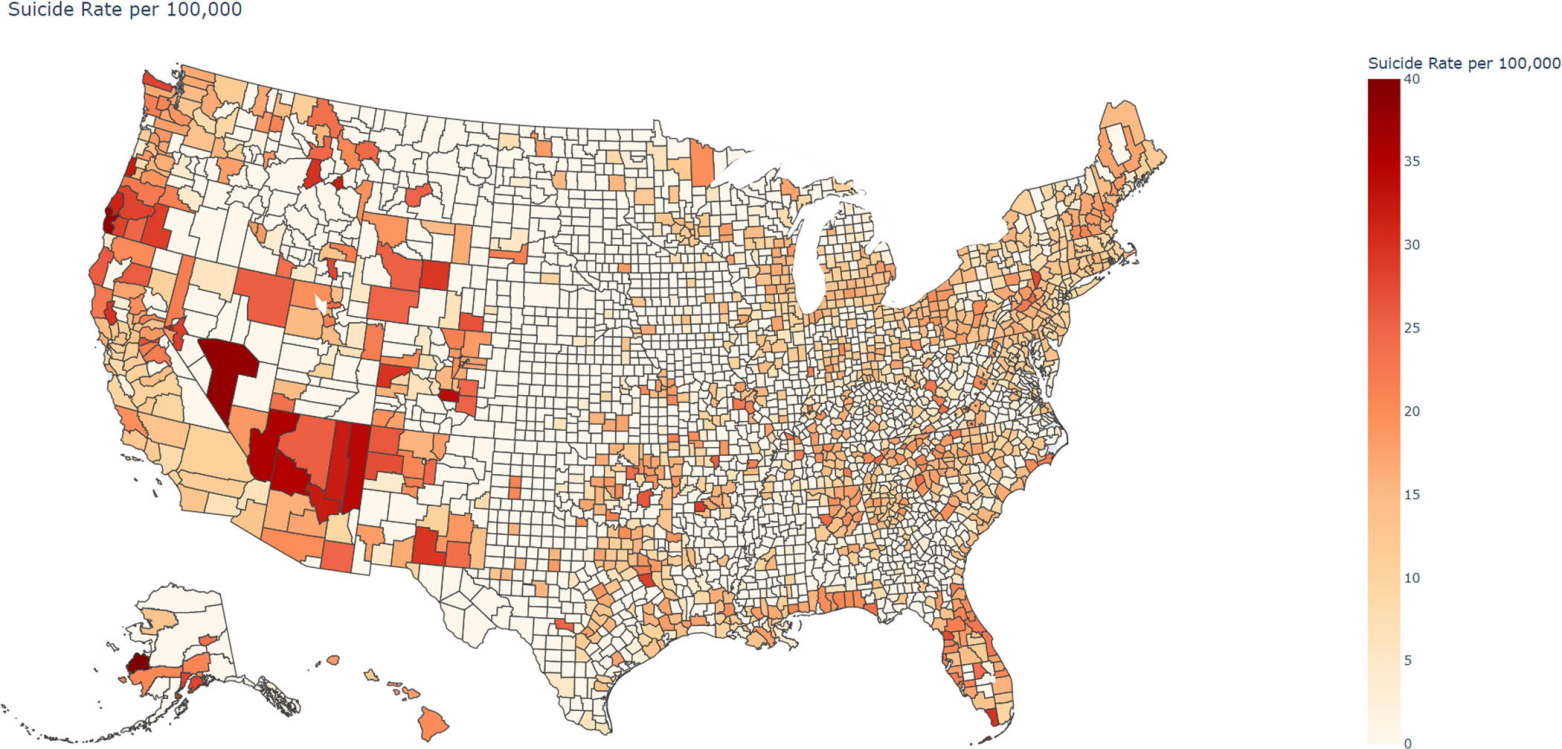
Average County level suicide rate per 100,000 from 2010 to 2019. Counties with low average suicide rate per 100,000 (range 0–10) are shown in light shade and the counties with high suicide rates (≥35) are displayed in dark shade.

The total number of suicides among US residents was found to be 365,286 between the years 2010–2019, with majority occurring in the last 3 years (2017, 2018, 2019) of this study. Data showed that nearly 25% of the total counties experienced at least a 10% increase in suicides from 2010 to 2019, with 12% of total counties exhibiting an increase of at least 50%. Figure 2 shows the average county level suicide rate per 100,000 from 2010–2019. Figure 2 is created using Choropleth Maps of the Plotly Open-Source Graphing Library for Python^45^.

In Fig. 2, counties with low average suicide rate per 100,000 (range 0–10) are shown in light shade and the counties with high suicide rates (≥35) are displayed in dark shade. County wide suicide distribution revealed that counties with higher suicide rates were consistently located across the western part of the US. The highest suicide rates across the 10-year period were observed in parts of Alaska, Washington, Oregon, California, Nevada, Arizona, New Mexico, Colorado, Wyoming, and Montana. However, most of the West North Central States (Iowa, Kansas, Minnesota, Missouri, Nebraska, North Dakota, and South Dakota) saw lower suicide rates.

### Suicide prediction

Machine Learning based Suicide prediction was implemented using XGBoost Regressor^35^ in Python and the model performance was measured using the *R*^2^ scores. The model with 17 unique features for each of the 3140 counties in the US predicted suicide rates with a *R*^2^ value of 0.98

### Feature importance

**Fig. 3:**
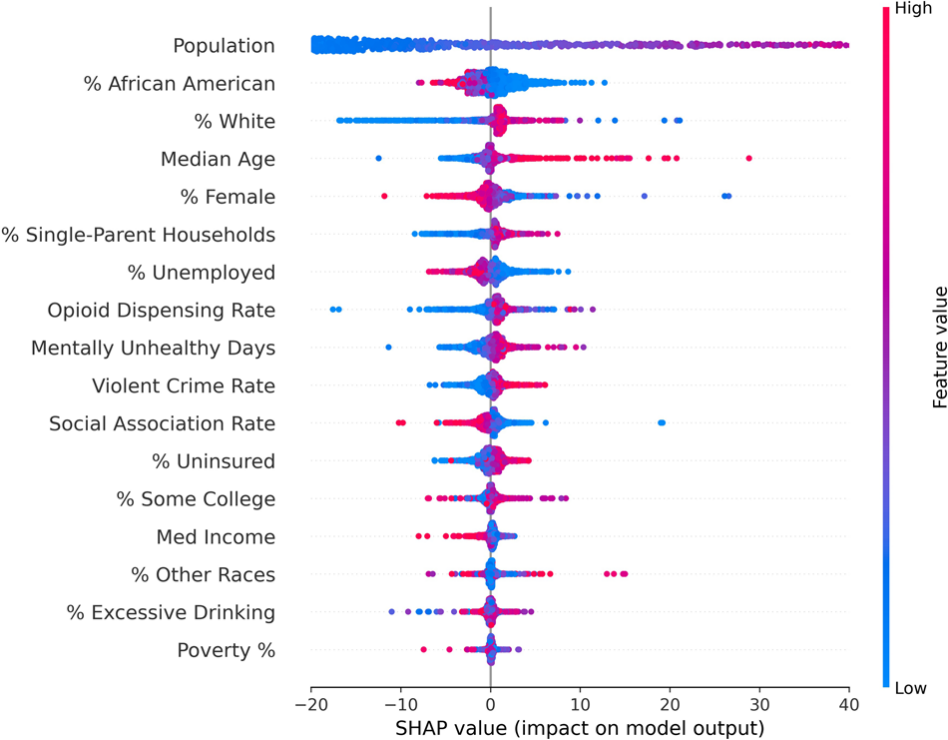
SHAP summary plot for the XGBoost-based suicide prediction model. SHAP summary plot reveals the relative impact of each feature on the prediction results.

The prediction model used in this work included 17 county factors as inputs. To identify the impact of each of these 17 features on the prediction results, the SHAP feature importance values were used^46^. SHAP values represents the contribution by each feature on a log-odds scale (the logarithm of the ratio of high suicides to low suicide). Figure 3 shows the SHAP value plot for all the 17 features used within the training dataset.

In Fig. 3, each of the 17 features supplied to the model are listed on the y-axis and arranged based on their importance. The variables on the top are the ones that affect the prediction results significantly (more importance) and those at the bottom are the ones that have less impact on the outcome (least importance). From Fig. 3, it can be inferred that the top 5 county level features significantly driving the prediction results are *Population* (Total Population of a county), *% African American Population*, *% White Population*, *Median Age*, and *% Female Population*. The x-axis shows the SHAP values corresponding to each feature. It is worthwhile to note that the influence of each case to the predicted output is represented by a specific dot in the figure. The color of the dots indicates if that corresponding feature value was high (Red) or low (Blue). The interpretation of Fig. 3 was performed based on the SHAP documentation^46^. The interpretations revealed that Population, % White Population, and Median Age had positive impact on suicide rate whereas features such as % African American Population, and % Female Population had negative impact on suicide rate.

To give the readers a glimpse of the interpretation process, two interpretations examples of Fig. 3 are discussed below using “Median Age” and “% African American Population” respectively as the county level feature of interest.

**Interpretation Example 1**: In Fig. 3, a “high” value for the median age will have a “positive” effect on the suicide prediction. The “high” value is denoted by the red color and the “positive” effect is inferred from the positive values in the x axis corresponding to the red dots. This means that as median age increases in a county, it tends to push the suicide values to the positive side, in that county. On the other hand, the “low” value for median age (blue dots) will have a “negative” effect (value corresponding to blue dots in the x-axis) on the suicide prediction. In other words, as median age decreases in a county, it tends to push the suicide values to the negative side, in that county.

**Interpretation Example 2:** In Fig. 3, a “low” value for the % African American population will have a “positive” effect on the suicide prediction. The “low” value is denoted by the blue color and the “positive” effect is inferred from the positive values in the x axis corresponding to the blue dots. This means that as % African American population decreases in a county, it tends to push the suicide values to the negative side, in that county. On the other hand, the “high” value for % African American population (red dots) will have a “negative” effect (value corresponding to blue dots in the *x*-axis) on the suicide prediction. In other words, as % African American population decreases, it tends to push the suicide values to the positive side, in that county.

### SVI

**Fig. 4:**
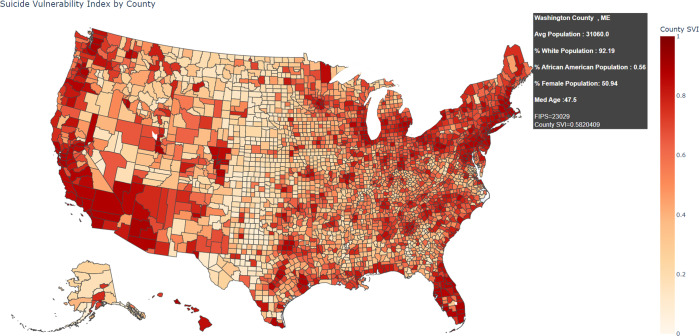
SVI distribution for US counties. An interactive version of this map, which reveals detailed county level statistics including median age, suicide rate, population etc. when hovered over a region, can be explored by downloading the Supplementary Fig. 1.

The top 5 features that significantly affected the prediction results which were identified in the previous section were used to create a novel SVI. The SVI score, calculated on a scale of 0-1 using the percentile contribution of the top 5 significant features, measures the resilience of a county towards suicides. In other words, counties with high SVI score are prone to a higher suicide rate than the counties with a low SVI score. The SVI distribution for the US counties is shown in Fig. 4 and an interactive version of the map can be explored by downloading the Supplementary Fig. 1. Both Fig. 4 and Supplementary Fig. 1 are created using Choropleth Maps of the Plotly Open-Source Graphing Library for Python^45^.

In Fig. 4, counties with high suicide vulnerability (in the range 0.8–1) are shown in a darker shade and the ones with low suicide vulnerability (in the range 0–0.2) are shown in a lighter shade. The county wide suicide vulnerability distribution revealed that counties with a high SVI were consistently observed in parts of Washington, Oregon, California, Nevada, Arizona, Florida, North Carolina, New York, Pennsylvania, New Jersey, Washington DC, and Michigan. Counties in the West North Central States (Iowa, Kansas, Minnesota, Missouri, Nebraska, North Dakota, and South Dakota) saw low SVI values. Table 2 lists 5 counties each with high SVI and low SVI, along with their corresponding percentile score for each of the 5 important county level features.

**Table 2:** Selected counties and their SVI.

County	State	Pop Pct	Age Pct	White Pct	AA Pct	Female Pct	SVI
High SVI							
Orange County	CA	1.00	0.22	0.08	0.54	0.49	0.90
Salt Lake County	UT	0.99	0.05	0.32	0.54	0.74	0.90
Pinellas County	FL	0.98	0.90	0.36	0.25	0.07	0.90
Suffolk County	NY	1.00	0.19	0.04	0.48	0.76	0.90
Santa Clara County	CA	0.99	0.50	0.29	0.30	0.37	0.90
Low SVI							
Beckham County	OK	0.02	0.16	0.43	0.49	0.97	0.08
Conway County	AR	0.02	0.69	0.51	0.26	0.38	0.08
New Madrid County	MO	0.01	0.67	0.48	0.19	0.05	0.06
Jones County	TX	0.01	0.42	0.17	0.24	0.99	0.06
Todd County	SD	0.01	0.01	0.01	0.94	0.25	0.05

*Pop Pct* Total Population Percentile Score; *Age Pct* Median Age Percentile Score; *White Pct* White Population Percentile Score; *AA Pct* African American Population Percentile Score; *Female Pct* Female Population Percentile Score.

## Discussion

Suicide is a serious public health concern in the US and is one among the leading causes of death. With the rate of suicides increasing rapidly every year, it is necessary to implement targeted suicide control and prevention efforts. This work examined suicide trends in all the US counties during the 10-year period between 2010–2019. The analysis showed that a vast majority of the counties experienced an increase in suicide rates over the 10-year period of study. It was also found that the counties in the coastal regions consistently ranked high in the number of suicides reported each year. This underscores the need to implement targeted suicide control and prevention efforts. The machine learning model developed to predict suicides was trained using 17 unique county level features such as demographics, socioeconomic factors, health related factors, and was found to perform with high accuracy in providing strong insights regarding county level suicide rates. The model can serve as a tool to monitor suicide rates in US counties and can be used as a foundation for future research in this field.

With the help of SHAP feature importance values, the impact of all the 17 factors on the prediction was analyzed and the top 5 important factors were identified. The SHAP plots revealed that county level features namely, Population, % White Population, Median Age had a significant positive impact on the suicide prediction rates and the % African American Population and % Female Population had a significant negative impact on suicide predictions. The SVI developed using the top 5 important county level factors revealed the counties prone to high suicide rates. The SVI distribution shows that population and suicide rates have a positive correlation or in other words, as the population in a county increase, the suicide rates generally increase. It can be observed that even though these tend to be in the eastern and western coastal areas, they are spread out across different states and do not necessarily belong to one single state. Moreover, it can be seen that even within a state, for instance Texas, there can be significant variation in the SVI values among its counties. This underlines the argument that a state or national level suicide trend may not necessarily reflect the trends of a particular region or geographic area. In this regard, the SVI developed for a county (which is the smallest possible geographical classification available in the CDC Wonder Database) can be used to identify the nuances within small regions and thus will be helpful in implementing targeted suicide control and prevention efforts.

**Fig. 5:**
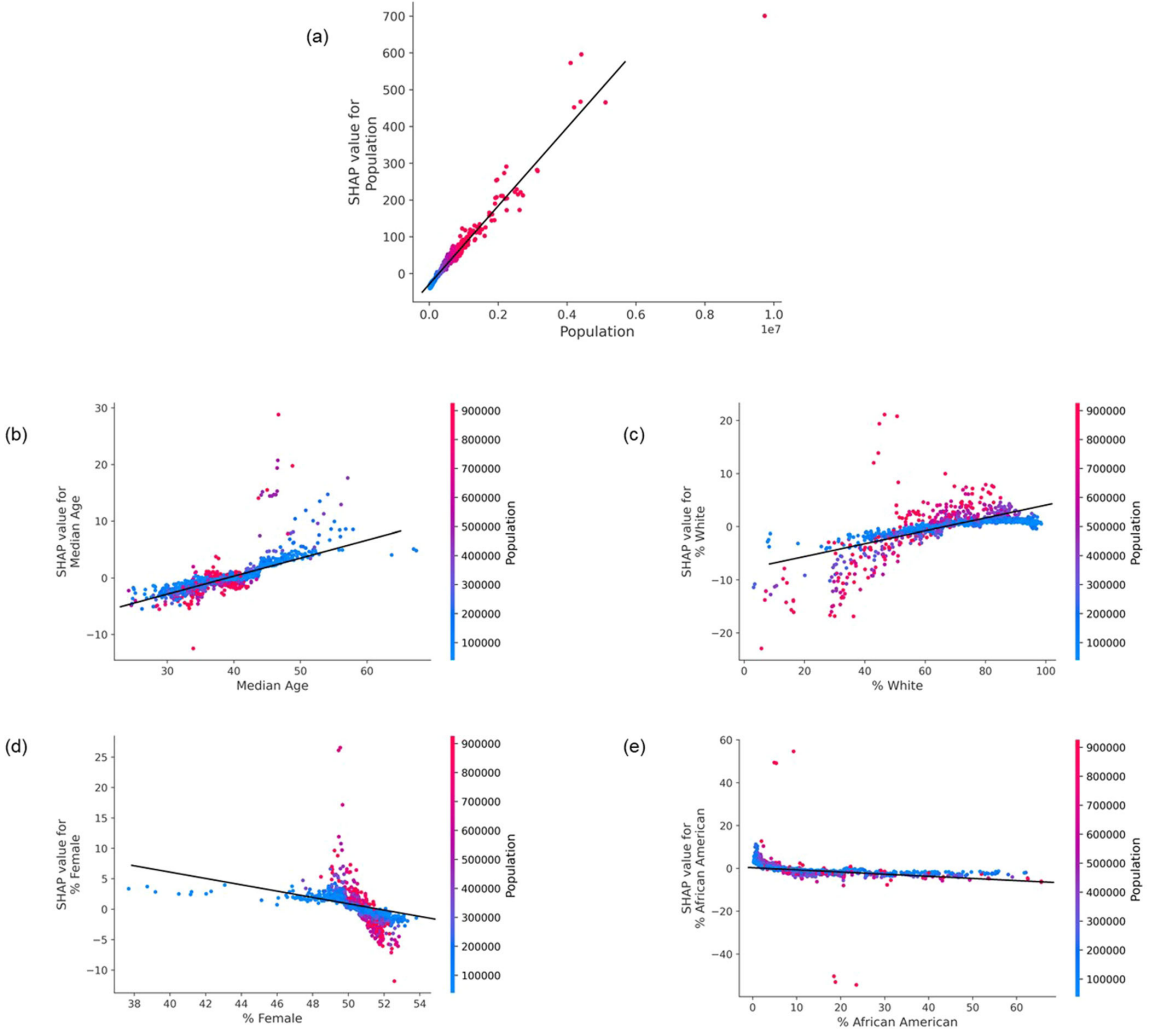
Dependency plot for top 5 important county level features. **a** Population, **b** Median age, **c** % White, **d** % Female, **e** % African American.

The interpretation results obtained from Fig. 3 regarding the effect (positive or negative) of each of the 5 important features used in the SVI development namely: *Population*, *% African American Population*, *% White Population*, *Median Age*, and *% Female Population* can be validated by generating a SHAP Dependence Plot^47^. The SHAP dependence plot can illustrate the effect of one specific feature/variable across the entire dataset. It essentially plots the feature’s value vs. it’s corresponding SHAP score^47^. As the SHAP score for a particular feature value becomes more positive, the feature tends to push the prediction result to the positive direction and vice-versa. The dependence plot for the top 5 significant features along with its interaction effect with the feature “Population” are shown in Fig. 5.

If we focus on Fig. 5a “Population”, the horizontal location depicts the actual value from the dataset and the vertical location shows the impact of a particular value on the prediction. The upward slope in Fig. 5a implies that as the “Population” increases, the model’s predicted value of suicide rates also increases (positive correlation). Similar observations can be made from both Fig. 5b “Median Age” and Fig. 5c “% White”. On the other hand, Fig. 5d “% Female” and Fig. 5e “% African American” shows a negative correlation. The downward slope in these cases imply that as these features increase, the model’s prediction value of suicide rates decreases. This matches and validates with the interpretation result obtained from Fig. 3 in the Result Section. Finally, the color coding gives an idea of the total population value of a county corresponding to each point in the data.

The SVI developed in this work was validated with the help of three unique contexts: (i) Comparison of the SVI of a county with its population to establish the claim that counties with a higher SVI are in counties with a higher population (ii) Comparison of SVI with county level Suicide Ideation (mental thoughts of committing suicide or self-harm) rates, and (iii) Comparison of SVI distribution with the county level suicide rate distribution, to assess whether SVI can accurately identify counties prone to high suicide rates. These are explained in detail below.

**Instance 1:** The SHAP values and the SHAP summary plot discussed in Results Section indicate that “Population” is the most important feature that drives the prediction result and has a positive impact. These results imply that the counties with larger population tend to have higher suicide rates which means, their SVI values should be high (in the range 0.8–1.0). To validate this hypothesis, the top 10 populous counties along with their suicide rates (averaged over the 10-year period from 2010–2019) were identified. It can be observed that the SVI for each of these counties is greater than or equal to 0.87, signifying high suicide risk. Table 3 lists the 10 populous counties along with their SVI.

**Table 3:** Top 10 populous counties and their SVI.

County	State	Average population	Average suicides	SVI
Los Angeles County	CA	9886598	834	0.88
Cook County	IL	5125064	437	0.87
Harris County	TX	4402240	457	0.88
Maricopa County	AZ	4073770	658	0.89
San Diego County	CA	3159234	414	0.89
Orange County	CA	3091453	323	0.90
Miami-Dade County	FL	2617239	249	0.87
Kings County	NY	2561987	134	0.86
Dallas County	TX	2491889	267	0.87
Riverside County	CA	2307365	260	0.89

**Instance 2:** According to the report^48^ from Mental Health America (MHA), US counties namely Los Angeles County (CA), Maricopa County (AZ) and Cook County (IL) recorded the more than one thousand individuals reporting thoughts of suicide or self-harm (Suicide Ideation). This list is followed by San Diego County (CA) and Orange County (CA) with over 700 individuals reporting suicide ideation. It can be observed that the SVI for each of these counties is greater than or equal to 0.87, signifying high suicide risk (High Risk when SVI is in the range 0.8–1.0). Table 4 lists the counties with high suicide ideation rates along with their SVI.

**Table 4:** Counties with high suicide ideation rates and their SVI.

County	State	Suicide ideation rate	Average suicides	SVI
Los Angeles County	CA	2496	834	0.88
Maricopa County	AZ	1289	658	0.89
Cook County	IL	1226	437	0.87
San Diego County	CA	801	414	0.89
Orange County	CA	725	323	0.90

**Instance 3:** The SVI generated using the top 5 important county level features indicated that most of the counties in the eastern and western coast and Florida region were susceptible to high suicide rates (Fig. 4). This observation agrees with the increasing suicide trend in these areas over the last decade as identified in Fig. 2 in the “Trends and Geographical Distribution of Suicide” section of the paper.

Thus, it can be concluded that indexes such as the SVI can help identify counties or regions prone to high suicide rates and may prove to be useful, especially during disasters and pandemics such as COVID-19, to effectively allocate resources and provide the necessary help and support. More importantly, this calls for the attention of researchers, policy makers and public health leaders for further investigation in the areas to boost the efforts for controlling and preventing suicides.

There are some limitations identified with this work. Firstly, it is possible that some suicide deaths in a county may not be actually classified as suicides due to the lengthy procedures attached to the process of identifying cause and mode of death. Secondly, the data obtained from CDC might not account for the suicides that went unreported. Moreover, unintentional self-harm leading to death is not usually classified as suicides, however, there might be cases where the self-harm leading to death was intentional, leading to underestimation of suicide deaths. Even though the study investigated suicide rates by counties, which is the smallest possible geographical classification available in the CDC Wonder Database, there might be variations within the county, which this work could not identify. Finally, despite the fact that this work tried to capture all possible features impacting the suicide rates, staying within the constraints of data availability, there can be other features which could impact suicide rates and were not included in the prediction model. For instance, this work does not separate out counties that have tribal populations, and therefore the effect of such populations on suicide rates is not isolated. The degree to which all these might happen, however, is unknown.

### Supplementary information

Supplementary Figure 1

## Data Availability

The datasets generated during and/or analyzed during the current study are available from the corresponding author on reasonable request.

## Code availability

The codes used for the generation and analysis of the dataset are available from the corresponding author on reasonable request.
